# Tumeurs stromales gastro-intestinales: une étude rétrospective

**DOI:** 10.11604/pamj.2023.45.97.36563

**Published:** 2023-06-21

**Authors:** Habib Zidane, Zakia Kebbati, Mohamed Khettab

**Affiliations:** 1 Service d’Oncologie Médicale, Centre Hospitalier Universitaire de Mostaganem, Faculté de Médecine de Mostaganem, Mostaganem, Algérie,; 2 Service d´Hémato-Oncologie, Centre Hospitalier Universitaire de la Réunion, Groupe Hospitalier Sud Réunion, Saint-Pierre, France

**Keywords:** Tumeurs stromales gastro-intestinales, diagnostic, traitement, évolution, Gastrointestinal stromal tumors, diagnosis, treatment, progression

## Abstract

**Introduction:**

bien que rares, les tumeurs stromales gastro-intestinales (GIST) constituent les tumeurs mésenchymateuses les plus fréquentes du tube digestif. Le but de ce travail est d´étudier les aspects cliniques, paracliniques, thérapeutiques et évolutifs des GIST.

**Méthodes:**

étude rétrospective descriptive, monocentrique (avril 2017-avril 2021) colligeant tous les patients ayant un GIST traités au niveau du service d´oncologie médicale du CHU de Mostaganem.

**Résultats:**

notre étude a colligé sur une période de 4 ans, 23 patients, avec un âge médian de 54,4 ans, sex ratio de 1,8. La douleur abdominale était le symptôme le plus fréquent (78,3%, n=18), la tumeur se localisait dans 47,8% des cas (n=11) au niveau de l’intestin grêle. Le diagnostic a été fait à un stade précoce dans 69,6 % des cas (n=16). Le traitement chirurgical a été fait chez 20 patients sur les 23 dont 18 avec R0. Des 15 patients opérés ayant un stade localisé; 13 ont reçu un traitement médical en adjuvant (Imatinib). L´évolution sous imatinib a été marquée par 3 progressions où on est passé vers une 2^e^ ligne (Sunitinib). Durant la période de l´étude tous les patients sont vivants sauf deux qui sont décédés après progression de la maladie.

**Conclusion:**

le diagnostic des GIST repose principalement sur l'histologie et l'immunohistochimie qui est souvent non faite par nos pathologistes. La biologie moléculaire permet de prédire le pronostic et en conséquence adapter les thérapeutiques. L´évolution des GIST est souvent favorable mais marquée par les récidives malgré un traitement supposé curatif imposant une surveillance prolongée.

## Abstract (en)

**Introduction:**

although rare, gastrointestinal stromal tumors (GIST) are the most common mesenchymal tumors of the digestive tract. The aim of this work is to study the clinical, paraclinical, therapeutic and evolutionary features of GIST.

**Methods:**

we conducted a descriptive retrospective, monocentric study (April 2017-April 2021) collecting data from the medical records of all patients with GIST treated at the medical oncology department of the University Hospital of Mostaganem.

**Results:**

we collected data from the medical records of 23 patients, with a median age of 54.4 years, sex ratio 1.8, over a period of 4 years. Abdominal pain was the most frequent symptom (78.3%, n=18); 47.8% of patients (n=11) had a tumor in the small bowel. The diagnosis was made at an early stage in 69.6% of cases (n=16). Surgical treatment was performed in 20 of the 23 patients, 18 of whom with R0. Of the 15 operated patients with a localized tumor, 13 received adjuvant medical treatment (Imatinib). Disease progression was reported in three patients treated with imatinib, then 2^nd^ line therapy (Sunitinib) was started. During the study period, all patients were alive except two who died due to disease progression.

**Conclusion:**

the diagnosis of GIST is mainly based on histology and immunohistochemistry, which is often not performed by our pathologists. Molecular biology makes it possible to predict the prognosis and consequently adapt the therapies. The outcome of patients with GIST is often favorable but marked by recurrences despite a supposedly curative treatment requiring prolonged monitoring.

## Introduction

Les tumeurs stromales gastro-intestinales (GIST) sont des tumeurs conjonctives rares, localisées généralement au niveau de l´estomac (60%) ou le grêle (30%), rarement au niveau du duodénum (4% à 5%) ou le rectum (4%) et exceptionnellement au niveau de l´œsophage, le côlon ou l'appendice (1 à 2%) [1]. Elles sont dérivées des cellules de Cajal ou de l'un de leurs précurseurs et expriment typiquement les phénotypes KIT+ (95% des cas) et DOG-1+ (95% des cas). Des mutations oncogéniques du gène KIT ou *platelet derived growth factor receptor alpha* (PDGFRA) codant pour le récepteur de la tyrosine kinase sont retrouvées dans environ 85% des GIST de l´adulte [2]. Ces mutations constituent des facteurs pathogènes importants qui induisent l'activation des protéines KIT ou PDGFRA. Le GIST est une tumeur rare, ne représentant qu'environ 1% des tumeurs de l'appareil digestif, son incidence est estimée à environ 15 cas/million d'habitants/an, l'âge médian au diagnostic est d'environ 60 ans, et le sexe ratio est d'environ 1/1 [3]. En Algérie, à l´exception des quelques résultats des différentes séries rétrospectives; les données sur les GIST sont encore insuffisantes. Le potentiel de malignité de ces tumeurs est bien connu et leur pronostic est corrélé à la localisation, la taille de la tumeur et l'index mitotique. Pour les formes localisées la chirurgie est le traitement de référence. A l´image des autres cancers, le développement des différentes techniques de séquençage a permis de comprendre les processus de carcinogénèse de ces tumeurs et par conséquence l´avènement de certaines molécules tels les inhibiteurs de récepteurs tyrosine kinase (ITK): imatinib puis sunitinib et regorafenib et qui ont bouleversé le pronostic des GIST en situation métastatique. La rareté de cette tumeur et le manque de données nous ont donc incité à mener cette étude. Le but de ce travail est de décrire les aspects épidemiologiques, cliniques, paracliniques, thérapeutiques et l´évolution des patients atteints de GIST dans notre centre.

## Méthodes

**Cadre et type de l´étude:** il s´agit d´une étude rétrospective descriptive, monocentrique, qui porte sur 23 cas consécutifs de GIST pris en charge au niveau du service d´oncologie médicale du centre hospitalier Universitaire de Mostaganem, sur une période de 4 ans allant du 1^er^ avril 2017 au 1^er^ avril 2021. Nous avons utilisé les mots clés suivant: Tumeurs stromales gastro-intestinales, diagnostic, traitement, évolution. Une recherche approfondie dans les bases de données PubMed et Google Scholar a été effectuée pour trouver les sources bibliographiques que nous permettront de discuter les résultats trouvés et rédiger cette étude.

**Participants à l'étude:** le recueil des données était effectué à partir des dossiers patients du service d´oncologie médicale et de service du chirurgie pour l´ensemble des dossiers inclus durant la période de l´étude. Pour chaque patient nous avons recueilli les données suivantes: âge au diagnostic, le sexe, le tableau clinique inaugural, la localisation, les caractéristiques anatomopathologiques et immunohistochimiques, les thérapeutiques reçues et les aspects évolutifs de la maladie. Une fiche d´exploitation a été untilisée pour la collecte des données de chacun des dossiers inclus. Le nombre de patient à inclure dans notre étude n´était pas défini au depart; une liste a été établie à partir du système informatisé au niveau de la salle d´archive du service d´oncologie médicale du centre hospitalier universitaire.

**Critères d'inclusion:** a) âge 18 ans et plus; b) cas de GIST confirmé par histologie; c) les patients pris en charge au niveau du Centre Hospitalier Universiataire de Mostaganem.

**Critères d'exclusion:** a) absence de preuve histologique d´un GIST; b) les patients ayant des dossiers médicaux non retrouvés.

**Analyse des données:** la saisie et l´analyse des données ont été faites en utilisant le logiciel statistique pour les sciences sociales (IBM Corp, Armonk, NY, États-Unis; SPSS Software version 21). Les fréquences et les pourcentages ont été utilisés pour rapporter les variables catégorielles, les variables quantitatives ont été exprimées en moyennes avec valeurs extrêmes. Les variables qualitatives ont été exprimées en effectifs et pourcentages.

## Résultats

**Figure 1:**
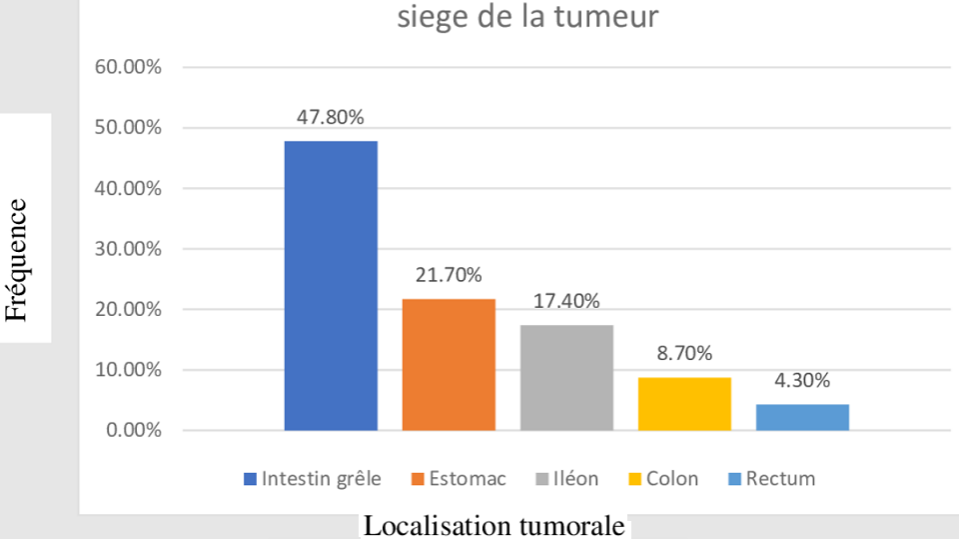
siège de la tumeur

Notre étude a identifié sur une période de 4 ans, 26 patients ayant un GIST, nous avons exclu 3 patients en raison de l´indisponibilité de leurs dossiers. Nous avons réalisé des analyses pour 23 patients avec un âge moyen de 54,4 [30-84 ans] ans, sex ratio de 1,8 (15 hommes pour 8 femmes), la douleur abdominale était le symptôme le plus fréquent (78,3%, n=18) (Tableau 1), l´intestin grêle était le site le plus fréquent de la tumeur (47,8%, n=11), suivi par l´estomac chez 5 patients (21,7%), l´ileon (17,4%, n=4), le colon chez deux patients (8,7%), le rectum (n=1, 4,3%) (Figure 1). Le diagnostic a été fait à un stade précoce dans 69,6% des cas (n=16), on avait quatre tumeurs localement avancées (17,4%) et trois en situations métastatiques (13%). Le diagnostic histologique a été posé dans 78,3% des cas par abord chirurgical (n=18), la biopsie sous endoscopie a permis de confirmer le diagnostic chez 5 patients (21,7%), la principale forme histologique était le GIST fusiforme (95,6%, n=22), un cas était épithélioide, l´immuno- histochimie a été faite chez seulement 11 patients (47,8%) dont 8 avaient un c-KIT positif. La taille moyenne de la tumeur était de 8,7 cm (4-26 cm), l´index mitotique a été calculé chez 10 patients (43,5%) dont 6 avaient un index ≥ 5, l´évaluation du risque évolutif selon la classification histo-pronostique établie lors d´un consensus en 2002 par Fletcher *et al*. [4] a permis de classer les tumeurs en des tumeurs à faibles risques de récidive chez un patient (4,3%, n=1), à risque intermédiaire chez 13 patients (56,5%) et à haut risque de récidive chez 7 patients (30,4%). Le traitement chirurgical a été fait chez 20 patients sur les 23 (86,9%) dont 18 (90%) avec R0.

**Tableau 1:** caractéristiques des patients

Variable	Effectif	Pourcentage
**Âge**	54,4 ans (30-84)	
**Sexe**		
Homme	15	65,2 %
Femme	8	34,8 %
**Motif de consultation**		
Douleur abdominale	18	78,3%
Troubles de transit	1	4,3%
Hémorragie digestive	1	4,3%
Syndrome occlusif	2	8,7%
Autre	1	4,3%
**Siège de la tumeur**		
Intestin grêle	11	47,8%
Estomac	5	21,7%
Iléon	4	17,4%
Colon	2	8,7%
Rectum	1	4,3%
**Stadification clinique (à l´admission)**		
Localisé	16	69,6%
Localement avancé	4	17,4%
Métastatique	3	13%
**Diagnostic positif**		
Biopsie	5	21,7%
Abord chirurgicale	18	78,3%
**Type histologique**		
GIST fusiforme	22	95,6%
GIST épithélioide	1	4,4%
**Immunohistochimie**		
C Kit +	8	34,8%
C Kit -	3	13%
Taille tumorale moyenne	8,7 cm (4-26)	
**Index mitotique**		
≥5	6	26,1%
<5	4	17,4%
Risque évolutif (C.Fletcher *et coll*.)		
**Faible risque**	1	4,3%
Risque intermédiaire	13	56,5%
Haut risque	7	30,4%

Le geste de résection était adapté à la localisation tumorale et à son extension locorégionale. Deux patients (10%) ont été opérés après avoir reçu un traitement néoadjuvant (Imatinib). Des 15 patients opérés R0 ayant un stade localisé; 13 ont reçu un traitement médical (Imatinib) en adjuvant (86,6%) (un patient présentait un GIST colique localisé n´a pas subi de chirurgie), il s´agissait de tumeurs à risque intermédiaire ou élevé de récidive, pour une durée de 3 ans pour les GIST à haut risque et 1 an pour les GIST à risque intermédiaire, deux patients en ont bénéficié en néoadjuvant pendant 6 à 12 mois pour des tumeurs jugées non résécables (un patient avec un cancer de l´estomac sous forme d´une masse de 10 cm de diamètre et un patient avec une tumeur iléale de 7 cm de diamètre). En situation métastatique (3 patients); l´imatinib a été indiqué en première ligne avec une dose de 400 mg/j. L´évolution sous imatinib a été marqué par 3 progressions (une après 4 cures, la 2^e^ après 11 cures et la 3^e^ après 17 cures) ou on est passé vers une 2^e^ ligne (Sunitinib). En néoadjuvant nous avons eu deux réponses partielles au traitement dont un patient qui a été opéré après. Pour les patients avec des stades localisés nous avons enregistré une seule rechute locale chez un homme deux mois après la fin du traitement adjuvant. Durant la période de l´étude tous les patients sont vivants sauf deux qui sont décédés après progression de la maladie et dans un tableau d´altération profonde de l´état générale.

## Discussion

Les tumeurs stromales sont l'entité la plus fréquente des tumeurs mésenchymateuses du tube digestif, mais sont considérées comme rares car elles ne représentent que 10 % des sarcomes des tissus mous et moins de 1% des tumeurs gastro-intestinales malignes [5]. L´age moyen du diagnostic est de 55 ans [3,5]. Dans notre série, la moyenne d´âge était de 54,4 ans, ce qui rejoint les séries internationales, notamment maghrébines ou l´âge moyen variait entre 54,2 ans et 60,5 ans (Tableau 2). Il n'existe pas de prédominance nette de sexe, seules certaines études retrouvent une discrète prépondérance masculine avec un sex-ratio voisin de 1,5 [6-8]. Dans notre série nous avons enregistré une prédominance masculine avec un sex-ratio de 1,8 (15 hommes/8 femmes), un résultat proche de celui de la série marocaine de Bazine *et al*. [9] avec un sex-ratio de 2,28 (16 hommes/7 femmes), cela peut être expliqué par la taille limité de l´échantillon. Cliniquement les GIST demeurent longtemps asymptomatiques, jusqu'à ce qu´elles deviennent très volumineuses ou se compliquent, leur découverte peut être fortuite dans environ 20% des cas, notamment lors d´une endoscopie digestive haute, d´un examen d´imagerie ou d´une intervention chirurgicale, le saignement digestif est la circonstance de découverte la plus fréquente, le diagnostic est fait généralement dans le cadre d´une anémie ferriprive ou une hémorragie extériorisée, lorsque la tumeur est ulcérée. Les autres symptômes possibles sont des douleurs abdominales peu spécifiques, une masse palpable, ou des formes compliquées telles qu´une perforation ou un syndrome occlusif (plutôt pour les GIST du grêle). Dans la série retrospective de Miettinen *et al*. le saignement digestif était le symptôme révélateur le plus fréquent (54%, n=676), les autres symptômes étaient les douleurs abdominales (17%, n=209) et la découverte d´une masse abdominale (5%, n=64), la lésion était de découverte fortuite chez 18% des patients (n=220) [10]. Dans notre série, la symptomatologie révélatrice était la douleur abdominale chez la quasi-totalité des patients (78,3%, n=18), suivie du syndrome occlusif (8,7%, n=2) et d´une hémorragie digestive chez un seul patient, de même et dans la série de Taoufiq *et al*. [11] la douleur a constitué le maitre symptôme (51,8%, n=28).

**Tableau 2:** l´âge moyen du diagnostic selon les séries

Etudes	Age moyen
Bazine *et al*. (Maroc) [9]	54,2 ans
Taoufiq *et al*. (Maroc) [11]	55 ans
Pracucho *et al*. (Brésil) [12]	59 ans
Abdulkareem *et al*. (Nigeria) [13]	46 ans
Chan *et al*. (Chine) [14]	66,6 ans
Pratic *et al*. (Maroc) [15]	56 ans
Olfa *et al*. (Tunisie) [16]	60,5 ans
Samlani *et al*. (Maroc) [17]	59 ans
**Notre série**	**54,4 ans**

Les GIST peuvent se développer à n´importe où le long du tube digestif, avec une fréquence décroissante de l´estomac vers le rectum, leur siège est réparti comme suit: l´estomac (60-70%), l´intestin grêle (20-30%), le côlon et le rectum (5-10%), la localisation œsophagienne est de 1 %, et exceptionnellement au niveau du mésentère et du grand épiploon [12-15]. Dans notre série, le grêle occupait 47,8% des localisations (n=11), l´estomac 21,7 % (n=5) et l´iléon 17,4% (n=4), des résultats qui ne sont pas en accord avec la littérature même s´ils sont proches de ceux retrouvés par Olfa *et al*. [16] ou le grêle occupait 40% des cas (n=10). Le diagnostic de GIST est dans un premier temps présomptif, basé sur des éléments endoscopiques, écho-endoscopiques ou radiologiques, alors que la confirmation du diagnostic est uniquement histologique, dans les tumeurs localement avancées, le scanner est l'examen clé menant au diagnostic, le diagnostic des GIST gastriques, duodénaux ou colorectaux est généralement fait par endoscopie, les petites tumeurs du grêle ne sont souvent détectables qu´à l´entero-scanner ou entero-IRM, l´enteroscopie ou la vidéocapsule; dans notre série, la fibroscopie gastrique réalisée pour les 6 cas de tumeurs gastriques a retrouvé un aspect de masse sous-muqueuse dans 4 cas. La taille moyenne de la tumeur dans notre série était de 8,7 cm (4-26 cm), un résultat inférieur à celui trouvé dans les séries de Samlani *et al*. [17] (10 cm) et Taoufiq *et al*. [11] (12,5 cm). Dans une étude française, 15% des GIST diagnostiquées mesuraient moins de 2 cm de diamètre et 34% entre 2 et 5 cm [3]. La taille de la tumeur dans la série de Olfa *et al*. [15] variait entre 0,8 et 24 cm, 27% (n=7) avaient une tumeur dont la taille était comprise entre 2 et 5 cm. La TDM spiralé thoraco-abdomino-pelvienne est la référence dans le bilan d´extension des GIST, les métastases synchrones se voient dans 25 à 30 % des cas et touchent principalement le foie et le péritoine [18-20], 13% (n=3) des GIST étaient diagnostiquées au stade métastatique chez nos patients, le même résultat trouvé dans la série de Pratic *et al*. [15] (13,33%, n=4), ce taux est nettement plus important dans d´autres études africaines [20-22] et maghrébines [9,11] , et il est lié surtout à la difficulté d´accès aux structures sanitaires de référence.

La principale forme histologique dans notre série était le GIST fusiforme (95,6%, n=22), un cas était épithélioide; dans la littérature; il est rapporté que les GIST à cellules fusiformes représentent 70% des cas, dans environ 20% des cas, les cellules sont épithélioides, les autres variantes histologiques sont plus rares, de même dans les études maghrébines le taux des GIST à cellules fusiformes varie entre 73,9% et 100% des cas [9,11,17]. L´index mitotique dans notre série a été précisé chez 10 patients dont 6 avaient un index ≥ 5. La détermination de l´index mitotique (sur 5 mm^2^) est fondamentale pour évaluer le risque de récidive, à l´image de notre série; la détermination de l´index mitotique n´est pas toujours réalisée; dans la série de Taoufiq *et al*. [11] il n'a été mentionné que chez 33 patients sur un échantillon de 54 malades avec un index au-delà de > 5/50 dans 75,6% des cas, Olfa *et al*. [16] rapporte un taux de 42,3% des patients qui avaient un index mitotique compris entre > 1 et ou ≤ 5, l´index mitotique n´a pas été précisé que chez 4 patients, dans la série de Samlani *et al*. [17] le compte mitotique tumoral était inférieur ou égal à 5 mitoses/50 dans 7 cas et supérieur à 10 mitoses dans 4 cas. Dans notre série, l´évaluation du risque évolutif selon la classification histo pronostique établie lors d´un consensus en 2002 par Fletcher *et al*. [4] a permis de classer les tumeurs en des tumeurs à faibles risques de récidive chez un patient (4,3%, n=1), à risque intermédiaire chez 13 patients (56,5%) et à haut risque de récidive chez 7 patients (30,4%). Dans la littérature, les résultats sont différents selon les séries et selon la taille des échantillons, d´après Olfa *et al*. [15] ; les tumeurs à faibles risques de récidive représentaient 32% des cas (n=8), à risque intermédiaire 24% (n=6) et à haut risque 44% (n=11), dans la série de Taoufiq *et al*. [11] le risque de récidive a été élevé chez 72,2% (n=39) des patients et modéré dans 11,1% des cas (n=6). Dans les stades localisés le traitement à visée curative consiste en leur résection chirurgicale complète, l´objectif de l´exérèse est l´obtention de marges macroscopiques saines (R0), il est essentiel d´éviter une perforation peropératoire qui va entraîner une dissémination péritonéale et une survie similaire à celle des patients ayant eu une exérèse incomplète dans certaines études, ces lésions souvent nécrotiques sont fragiles et doivent donc être manipulées avec la plus grande précaution. À la différence des carcinomes, les GIST sont peu lymphophiles, le curage ganglionnaire ne doit donc pas être réalisé systématiquement, mais uniquement en cas d´atteinte ganglionnaire macroscopique [3,15]. Le traitement chirurgical dans notre série a été fait chez 20 patients sur les 23 dont 18 avec R0. Le traitement médical (Imatinib) a été fait en adjuvant dans 86,6% des cas opérés (n=13) pour des tumeurs classées à risque intermédiaire ou élevé de récidive, pour une durée de 3 ans pour les GIST à haut risque et 1 ans pour les GIST à risque intermédiaire.

La décision d´un traitement adjuvant doit se faire en fonction du potentiel de récidive de la tumeur (faible, intermédiaire, et haut risque de récidive) et en tenant compte des caractéristiques moléculaires qui déterminent la sensibilité potentielle a l´imatinib [3]. L´analyse histologique de la pièce opératoire est donc primordiale, ainsi que le génotypage. L´imatinib (inhibiteur de tyrosine kinases) a été testé en adjuvant dans l'essai multicentrique américain ACOSOG Z9001 qui a évalué la survie sans récidive chez plus de 700 patients atteints de GIST ≥ 3 cm randomisés pour recevoir de l'imatinib 400 mg/j ou un placebo pendant un an, la survie sans récidive était de 98% dans le bras imatinib contre 82% dans le bras placebo (p < 0,0001), sans bénéfice sur la survie globale et le sous-groupe à faible risque n'a montré aucun bénéfice à l'imatinib. Secondairement, le génotypage tumoral a permis d´observer une augmentation significative de la survie sans récidive uniquement pour les mutations de l´exon 11 (mais les autres sous-groupes sont d´effectifs plus faibles) [3]. L'essai multicentrique européen AIO-SSG a évalué la survie sans récidive d'environ 400 patients atteints de GIST perforé à haut risque de récidive [23], les patients étaient traités par imatinib pendant 1 ou 3 ans après la chirurgie, la survie sans récidive était de 66% versus 48% en faveur du traitement du bras 3 ans (p < 0,0001) (suivi médian de 54 mois), avec cette fois-ci un gain en survie globale: 92 % dans le bras 3 ans contre 82 % (p=0,019), après un suivi médian de 7,5 ans les résultats montrent que le bénéfice de l´imatinib 3 ans se maintient dans le temps, cette étude a permis à l´imatinib 400 mg/jour pendant une durée de 3 ans de devenir le standard thérapeutique en cas de GIST à haut risque de récidive. Il est important de noter qu´il n´y a pas d´intérêt au traitement adjuvant des GIST avec mutation D842V de l´exon 18 de PDGFRA, considérée comme une mutation de résistance à l´imatinib et associée à un risque de récidive spontané plus faible (accord d´experts) [3]. Dans notre série; deux patients ont bénéficié de l´imatinib en néoadjuvant. Un traitement néoadjuvant par imatinib est discuté dans des cas particuliers, ou l´objectif est la préservation d´organe et/ou l´augmentation du taux de résection R0 (downstaging) (par exemple GIST du bas rectum), soit de tumeurs de résécabilité borderline. Dans ce domaine, il n´y a pas d´étude randomisée ayant réellement évalué la place de l´Imatinib et les modalités de prescription, la dose la plus étudiée est 400 mg/j, avec une évaluation tous les 2-3 mois et d´opérer lorsque le volume tumoral est le plus faible, ou après une stabilité sur 2 imageries consécutives, après un traitement de l´ordre de 6 à 12 mois, qui permet d´obtenir un taux de réponse objective maximal [24]. Dans notre série; en situation métastatique (3 patients); l´imatinib a été indiqué en première ligne avec une dose de 400 mg/j.

L´imatinib est le seul traitement de première ligne avec une survie à un an d´environ 90% dès les premiers essais comparés à 40% avec les anciennes chimiothérapies qui ne sont pas efficaces [2]. Dans un essai randomisé de phase II chez 147 patients, une réponse partielle était observée dans 54% des cas, une stabilité dans 28%, soit plus de 80% de contrôle tumoral [25], l´efficacité de l´imatinib était maintenue chez environ 30% des patients à 5 ans et 20 % à 9 ans, le pronostic des patients était similaire en cas de réponse ou de stabilité. Il est important de noter que le statut mutationnel a un impact pronostique majeur: les survies sans progression et globales sont significativement meilleures en cas de mutation de l´exon 11 de KIT par rapport à une mutation de l´exon 9, elle-même de meilleur pronostic que les GIST wild-type, la posologie recommandée d´imatinib est de 400 mg/jour en continu jusqu'à progression ou intolérance, sauf en cas de mutation de l´exon 9 de KIT (800 mg/jour) [2]. L´étude BFR14 évaluant l´arrêt de l´imatinib comparé à sa poursuite chez des patients en réponse ou stabilité prolongées a montré que l´arrêt s´accompagne de reprises évolutives dans la grande majorité des cas, que l´imatinib soit arrêté après 1, 3 ou 5 ans de traitement [2,6] , ce même essai met en évidence que la reprise de l´imatinib en cas de progression ou récidive à l´arrêt de ce dernier permet de nouveau un contrôle tumoral chez 94% des patients ayant été mis en pause, sans effet négatif sur la survie globale, mais au prix d´un pronostic cependant plus péjoratif chez les patients ayant repoussé précocement et d´une réponse objective moins importante qu´initialement. La résistance primaire à l´imatinib, c´est-à-dire survenant dans les 6 premiers mois, est rare, on l´observe dans environ 5% des cas. Néanmoins il faut faire attention à la fausse progression radiologique, les GIST étant imparfaitement évaluées par les critères RECIST qui ne prennent pas en compte la variation de la densité des lésions liée à des phénomènes de remaniement tumoral et qui peut se traduire par une augmentation initiale de la taille [2]. Il est également indispensable de vérifier l´observance au traitement et de possibles interactions médicamenteuses (par exemple les inhibiteurs de la pompe à protons), en cas de progression avérée, doubler la posologie à 800 mg/jour permettait un nouveau contrôle temporaire de la maladie chez 30 à 40 % des patients [3].

Le Sunitinib est un ITK agissant sur plusieurs récepteurs tyrosine kinase transmembranaires (KIT, VEGF, PDGF) [26], il s´agit du seul ITK ayant une AMM en deuxième ligne, son efficacité a été démontrée par une étude de phase III multicentrique chez 312 patients avec une GIST métastatique ou non résécable ayant une résistance ou une intolérance à l´imatinib, la posologie classique est de 50 mg/j, 4 ON/ 2 OFF. Le regorafenib est un ITK qui inhibe de multiples protéines kinases impliquées dans l'angiogenèse tumorale (VEGFR 1, 2, 3, TIE2), l'oncogenèse (KIT, RET, RAF-1, BRAF, BRAF V600E) et le microenvironnement (PDGFR, FGFR). Il s´agit du seul ITK ayant une AMM en troisième ligne, en cas d´échec et/ou intolérance de l´imatinib et du Sunitinib, son efficacité a été démontrée en troisième ligne dans un essai de phase III après échec ou intolérance de l´imatinib et du Sunitinib et offrait une médiane de survie sans progression significativement augmentée (4,8 mois contre 0,9 mois dans le groupe placebo; p < 0,0001) et une survie globale non modifiée (il existait un cross-over) [27], la posologie recommandée est de 160 mg/ jour, 3 semaines sur 4. L´évolution sous imatinib dans notre série a été marquée par 3 progressions (la première après 4 cures, la 2^e^ après 11 cures et la 3^e^ après 17 cures) ou on est passé vers une 2^e^ ligne (Sunitinib). En néoadjuvant nous avons enregistré deux réponses partielles au traitement dont un patient qui a été opéré après. En adjuvant nous avons enregistré une seule rechute locale chez un homme deux mois après fins du traitement adjuvant. Durant la période de l´étude tous les patients sont vivants sauf deux qui sont décédés après progression de la maladie et dans un tableau d´altération profonde de l´état générale. Dans l´essai BFR14, la médiane de survie sans progression était de 30 mois et la médiane de survie globale de 6,4 ans [26].

**Limites**: nos résultats sont basés sur des données recueillies à l'hôpital. Les données indiquant un biais dû à un enregistrement incomplet ont été exclues car il était difficile de communiquer avec les patients pour un suivi à long terme ou même avec leurs médecins. La taille limitée de l´échantillon nous a empêché d'évaluer l'efficacité du traitement notamment en situation métastatique car nous avions que 3 patients qui ont été mis sous imatinib. Le manque de génotypage de la tumeur et particulièrement les mutations de l´exon 11 de Kit et l´exon 9 et celles du D842V de l´exon 18 de PDGFRA (qui sont des facteurs pronostics et prédictifs de la réponse à l´imatinib) nous a empêché d´évaluer l´efficacité de la molécule.

**Recommandations**: les GIST sont des tumeurs rares dont le pronostic depend de la qualité de la prise en charge initiale (diagnostic précoce, chirurgie carcinologique). Nous recommandons d'autres études avec des echantillons plus importants pour évaluer tous les aspects épidemiologiques, cliniques et thérapeutiques de la pathologie notamment les aspects evolutifs sous thérapies ciblées ou le genotypage tumorale joue un role trés important.

## Conclusion

Les GIST demeurent des tumeurs rares de l´adulte, majoritairement sporadiques. L´estomac est le siège le plus fréquent rapporté dans la littérature, dans notre série nous rapportant un taux 3 fois moins. Le génotypage tumoral des GIST manque toujours en Algérie notamment la mutation de l´exon 11 et celle du D842V de l´exon 18 de PDGFRA qui sont indispensables pour l´indication de l´imatinib. Les avancées spectaculaires de la biologie moléculaire, vont conduire certainement dans un futur proche à un traitement personnalisé et définitif des tumeurs stromales gastro-intestinales.

### Etat des connaissances sur le sujet

- *Les GIST sont des tumeurs rares localisées principalement au niveau de l´estomac et de l´intestin grêle;*
- *La biologie moléculaire joue un rôle important dans la prise en charge des GIST;*
- *Le pronostic s´est beaucoup amélioré après l´avènement de nouvelles classes thérapeutiques notamment les inhibiteurs de la tyrosine kinase (Imatinib)*.

### Contribution de notre étude à la connaissance

- *Dans la littérature; la symptomatologie révélatrice la plus fréquente est l´hémorragie digestive alors que dans notre série, était la douleur abdominale (78,3%);*
- *L´estomac est le siège le plus fréquent des GIST rapporté dans la littérature (60-70%), dans notre série nous rapportant un taux 3 fois moins (21,7%);*
- *Le génotypage tumoral des GIST manque toujours en Algérie notamment la mutation de l´exon 11 et celle du D842V de l´exon 18 de PDGFRA qui sont indispensables pour l´indication de l´imatinib*.
